# Anti-SARS-CoV-2 Antibody Screening in Healthcare Workers and Its Correlation with Clinical Presentation in Tertiary Care Hospital, Kathmandu, Nepal, from November 2020 to January 2021

**DOI:** 10.1155/2022/8515051

**Published:** 2022-02-01

**Authors:** Suraj Aryal, Sanskriti Pandit, Sushant Pokhrel, Mandira Chhusyabaga, Pabitra Bista, Mahendra Psd. Bhatt, Dharma Datta Subedi, Basista Psd. Rijal

**Affiliations:** ^1^Department of Laboratory Medicine, Manmohan Memorial Institute of Health Sciences, Kathmandu, Nepal; ^2^Department of General Medicine, Manmohan Memorial Medical College and Teaching Hospital, Kathmandu, Nepal; ^3^Department of Clinical Microbiology, Manmohan Memorial Medical College and Teaching Hospital, Kathmandu, Nepal

## Abstract

**Background:**

Antibody titer and the life span of antibodies against SARS-CoV-2 have been found to be associated with the clinical presentation in individuals. The extent of exposure of healthcare workers and the general public to SARS-CoV-2 needs to be assessed to monitor the COVID-19 pandemic. Thus, this study is an attempt in assessing the anti-SARS-CoV-2 antibody in health care workers.

**Methods:**

This laboratory-based cross-sectional study was performed in Manmohan Memorial Medical College and Teaching Hospital, Kathmandu from November 2020 to January 2021. A total of 185 HCWs were enrolled in this study. Their serum samples were screened for anti-SARS-CoV-2 antibodies, and a structured questionnaire was administered to collect further information. Anti-SARS-CoV-2 antibody screening was performed using lateral flow immunoassay. The data were analyzed using SPSS version 20.

**Results:**

Among 185 HCWs that participated in the study, 41 (22.2%) tested positive for the anti-SARS-CoV-2 antibody. Of these 41 HCWs, 37 tested positive for IgG only and 4 of them tested positive for both IgM and IgG antibodies. The presence of the previous history of SARS-CoV-2 infection (*p* < 0.001), the presence of flu-like symptoms within the last 6 months (*p* < 0.001), and the presence of positive contact history (*p*=0.002) were statistically significant with the presence of the antibody among HCWs.

**Conclusion:**

Healthcare workers carry a high burden of SARS-CoV-2 infection and are at risk of acquiring infection from their workplace. Anti-SARS-CoV-2 antibody screening among healthcare workers is highly recommended in multiple healthcare settings as it can help in monitoring transmission dynamics and evaluation of infection control policies.

## 1. Introduction

Severe acute respiratory syndrome coronavirus 2 (SARS-CoV-2) is an enveloped nonsegmented positive sense RNA *β*-coronavirus of the Coronaviridae family [1]. It was first diagnosed in December 2019 in Wuhan, China in a patient showing symptoms of atypical pneumonia [2]. The virus was found to cause a new disease called coronavirus disease 2019 (COVID-19) having high infectious potential [3]. Since the first case was identified in China, it has spread globally, and it has been declared a pandemic by the World Health Organization (WHO) on 11 March 2020. As of 24 June 2021, there have been 179,241,734 confirmed cases of COVID-19 globally, resulting in 3,889,723 deaths. On the same date, the total number of confirmed cases in Nepal has reached 629,431, with a total number of 8,918 deaths [4].

Most of the cases of COVID-19 are asymptomatic or with mild symptoms. Individuals characterized as high-risk groups (old aged, diabetic, hypertensive, and cancer patients, individuals with pulmonary diseases or disorders) were found to develop severe clinical presentations leading to multiple organ failure and death [5]. The principle of immune response and development of antibodies against SARS-CoV-2 is being studied globally. Infection with SARS-CoV-2 evokes the development of IgM and IgG antibodies, which is useful in the assessment of immune response following infection. These antibodies can be detected in the serum within 1 to 3 weeks after the onset of illness [6]. Antibody titer and the lifespan of antibodies against SARS-CoV-2 have been found to be determined by the clinical presentation as well. Short-lived and low antibody titers near the detection limit were found in the cases of mild infections [7].

Healthcare workers (HCWs) are considered a high-risk group for SARS-CoV-2 infection. They may acquire infection either from the healthcare settings or from the community. Several studies on the prevalence of SARS-CoV-2 antibodies among HCWs have reported the seroprevalence ranging from 1.6% to 45.3% [8]. According to the study performed by Poulikakos et al. in a tertiary care center in North West England, 6% of the healthcare workers tested positive for SARS-CoV-2 IgG antibody [9]. The study performed by Steensels et al. in Belgium showed that 6.4% of healthcare workers tested positive for SARS-CoV-2 IgG antibody [10]. The study performed by Mascola et.al.in New York showed that 13.7% of healthcare workers were seropositive for anti-SARS-CoV-2 antibodies [11]. Exposure to a large number of patients (either symptomatic or asymptomatic) in the hospital for a longer period may be the most common cause of infection for healthcare workers [8, 12]. Serological assays could be an important method for screening infections and epidemiological studies [13] and also for detecting symptomatic and asymptomatic infections [8]. Thus, this study aims to assess the seroprevalence of anti-SARS-CoV-2 antibodies among healthcare workers and to determine the association between clinical presentations and antibody screening in a tertiary care hospital in Kathmandu, Nepal. Furthermore, this study could be useful in assessing the exposure of HCWs to SARS-CoV-2 that will help to assess and monitor transmission dynamics and in the evaluation of infection control policies that would ultimately help in managing the COVID-19 pandemic. It is also recommended by international health authorities to screen for SARS-CoV-2 infection as it would be useful in reducing morbidity, transmission rate, and maintaining the health system within the balance.

## 2. Methods

A laboratory-based cross-sectional study was performed in Manmohan Memorial Medical College and Teaching Hospital (MMTH), Kathmandu, Nepal, during the period of 3 months (November 2020 to January 2021).

### 2.1. Selection of Study Population

HCWs, including administrative staff working at MMTH at least 6 months before the sample collection date, were invited to participate in the study voluntarily. The study was conducted in all departments of the hospital. All HCWs providing informed written consent were conveniently selected for the study and were requested to fill out the corresponding questionnaire to collect the required information, and HCWs who refused to give the informed written consent (*n* = 16) were excluded from the study. The professional category of the individuals participating in the study was categorized into three risk groups based on the degree of exposure to COVID-19 as [14]follows:

1. High-risk group: professionals working in a clinical environment with prolonged direct contact with patients (e.g., nurse, doctor)
2. Moderate risk group: professionals working in a clinical environment with nonintense/no direct patient contact but are at higher risk of nosocomial exposure (e.g., laboratory professionals, support staff)
3. Low-risk group: professionals working in a nonclinical environment with no patient contact or minimal patient contact (e.g., administrative staff)

### 2.2. Experimental Protocol

Blood samples were collected from HCWs after taking informed written consent by venipuncture following standard operating protocol. Samples were collected in a serum separator tube (HEBEI XINLE SCI & TECH CO.LTD, China) containing gel after obtaining informed and written consent.

Samples were centrifuged to obtain serum. Antibody screening was performed from the serum using SARS-CoV-2 IgM/IgG Antibody Assay Kit (Colloidal Gold Method) (Zybio Inc., China) as per the manufacturer's instruction (5 *μ*l serum was added to the sample well using a pipette, followed by the addition of 2 drops of buffer), the results were read within 10 to 15 minutes, and the results after 15 minutes were considered invalid.

The test was validated by positive and negative controls. The serum from the patient with a history of PCR confirmed SARS-CoV-2 infection within the last 3 weeks was used as a positive control, and the serum of a healthy individual without any contact history and any history of SARS-CoV-2 infection was used as a negative control.

### 2.3. Statistical Analysis

The data were collected in Microsoft Excel 2013 and analyzed using SPSS version 20.0 (IBM Corp., Armonk, NY, USA). Age was the only continuous variable defined using median and interquartile range (IQR). Mann–Whitney *U* test was used for assessing group differences in age. Categorical variables were expressed as frequency rates (*N*) and percentages (%). The chi-square test or Fisher exact test was used as applicable to test for association between categorical variables. *p* value <0.05 was considered statistically significant.

## 3. Results

**Figure 1:**
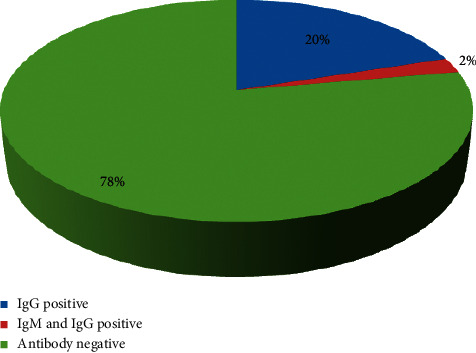
Antibody screening among HCWs.

A total of 185 HCWs participated in the study, and 41 (22.2%) of them tested positive for the anti-SARS-CoV-2 antibody. Among these 41 individuals, 37 tested positive for IgG antibody, while 4 tested positive for both IgM and IgG antibodies (Figure 1). All 4 individuals who tested positive for both IgM and IgG had a recent history of SARS-CoV-2 infection within a month.

The median age of HCWs was 27 (IQR 24–36) years, and 115 (62.1%) of them were in the age group of 20 years to 30 years. Overall, 52 (28.1%) male and 133 (71.9%) female HCWs participated in the study. Among the 41 individuals, 15 (36.6%) males and 26 (63.4%) females tested positive for the antibody.

The maximum number of individuals who tested positive for antibodies were nurses (39.0%), followed by laboratory professionals (19.5%), administrative staff (17.1%), doctors (14.6%), and supporting staff (9.8%), respectively (Table 1).

Of the total of 185 participants, 113 were of the high-risk group, and among them, 11.89% (22/185) had positive antibody screening. Similarly, 47 had moderate risk, and 25 were with low risk. The prevalence of antibody positivity was found to be 6.4% (12/185) in the moderate-risk group and 3.78% (7/185) in the low-risk group, respectively (Table 2).

Among the 185 individuals tested, 39 (21.1%) individuals had a previous history of PCR confirmed SARS-CoV-2 infection, and among those 39 individuals, only 21 of them tested antibody positive, whereas 18 of them tested negative for the antibody. All of the 18 subjects who tested negative for the antibody had an asymptomatic infection, and none of them had a history of severe illness.

**Figure 2:**
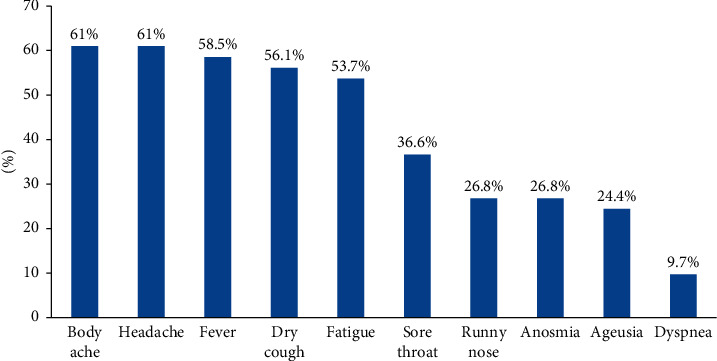
Clinical presentations among HCWs who tested positive for antibody.

Overall, 48 (25.9%) individuals had a history of flu-like symptoms within the last 6 months, and 27 of them were found to be antibody positive. Among 27 of the symptomatic antibody-positive individuals, body ache, headache, fever, dry cough, and fatigue were predominant symptoms, followed by sore throat, runny nose, anosmia, ageusia, and dyspnea (Figure 2).

All 41 individuals who tested positive for antibodies had positive contact history with the SARS-CoV-2 infected patients. The presence of the previous history of SARS-CoV-2 infection (*p* < 0.001), the presence of flu-like symptoms within the last 6 months (*p* < 0.001), and the presence of positive contact history (*p*=0.002) were statistically significant with antibody presence among HCWs (Table 1).

## 4. Discussion

COVID-19 is a global pandemic, infecting more than 179 million people around the globe, causing death to more than 3.8 million as of 24^th^ June 2021. It is believed that almost all immune-competent individuals after being infected with SARS-CoV-2 will develop an immune response against it [6]. HCWs are considered a high-risk group for SARS-CoV-2 infection. They may acquire infection either from the healthcare settings or from the community. Exposure to a large number of patients (either symptomatic or asymptomatic) in the hospital for a longer period may be the most common cause of infection for healthcare workers [8, 9].

The seroprevalence of antibodies against SARS-CoV-2 among HCWs in our study was 22.2%. The “Enhanced Surveillance on Seroprevalence of SARS-CoV-2 in General Population” of Nepal performed by the Government of Nepal, Ministry of Health and Population in collaboration with WHO in the second and third week of October 2020 found the seroprevalence in the general population to be 14.4% [15]. Hence, it showed that seroprevalence among HCWs is higher in comparison to the seroprevalence in the general population of Nepal. Similarly, the study performed by Varona et al. in over 6000 HCWs in Spain found higher seroprevalence in HCWs than in the general population [14].

Our study revealed significantly higher seroprevalence among healthcare workers compared to the previously published reports, ranging prevalence rates from 1.26% to 19.1%. A study performed by Psichogiou et al. in Greece found the seroprevalence rate to be 1.26% and mentioned the low burden of COVID-19 in Greece could be the reason for lower seroprevalence in the study [8]. The study performed by von Huth et al. in 7950 HCWs in Denmark found the seroprevalence rate to be 2.1%. [16]. Another study by Varona et al. in over 6000 HCWs in Spain found the seroprevalence rate to be 11.0% [14]. The study performed by Rudberg et al. in Sweden found the seroprevalence rate to be 19.1% [17]. A similar study by Lombardi et al. in Italy revealed the Seroprevalence rate to be 7.6% [18]. The higher seroprevalence in our study corresponds with the 24.4% prevalence rate as reported by Shields et al. in the UK [19].

Galanis et al., in their study, showed that the overall seroprevalence of anti-SARS-CoV-2 antibodies among HCWs was 8.7%, ranging from 0% to 45.3%. Higher seroprevalence was found in the studies conducted in North America (12.7%) as compared to the studies conducted in Europe (8.5%), Africa (8.2%), and Asia (4%) [20]. A similar study performed by Hossain et al. found a higher rate of seroprevalence in the USA (12.4%) compared to the seroprevalence rates in Europe (7.7%) and Asia (4.8%) [21]. The study performed by Houlihan et al. in London reported that 45.3% of HCWs were seropositive [22], and Vaselli et al. reported that the seroprevalence was higher among the HCWs in the UK compared to the HCWs in the rest of Europe during the months of March and August 2020 [23]. Likewise, the study by Müller et al. in the African countries found the seroprevalence to range from 0% to 45.1% [24]. Seroprevalence was higher in the study conducted in Nigeria (45.1%) compared with the study conducted in Libya (0%), Egypt (1.3%), and Togo (1.4%) [25–28]. Our study revealed relatively higher seroprevalence among HCWs as compared to the reports. The lack of programs for the regular screening of HCWs for SARS-CoV-2 infection, long-term exposure of HCWs with the infected cases (symptomatic or asymptomatic), and lack of proper PPE might explain the relatively higher seroprevalence in our study [29]. In the study performed by Lahner et al. in Italy, it was discussed that longer working hours, the improper use of personal protective equipment (PPE), and long-term exposure to patients could be the risk factors for HCWs for acquiring the disease [13].

In our study, 39.0% of seropositive HCWs were nurses, suggesting that the seroprevalence is relatively higher among nursing staff than in other HCWs. It could be because of the direct involvement of nursing staff in patient care and treatment, or it could be because of the higher number of nursing staff participating in this study in comparison to other HCWs. Similar to our study, Wilkins et al. in Chicago reported nurses as the highest risk group for obtaining infection [30], and a study performed by Al Maskari et al. in Oman reported that 38% of the infected HCWs were nurses [12].

Our study demonstrates higher seropositivity among the high-risk group and moderate risk group in comparison to the low-risk group, which is in accordance with the findings of Varona et al. in Spain, in which it is demonstrated that high-risk and moderate-risk groups presented a higher probability of seropositivity as compared to the low-risk group [14].

In the study population, 51.2% of seropositive HCWs in our study had a history of RT-PCR confirmed SARS-CoV-2 infection, and the remaining 48.8% never obtained positive RT-PCR results until the time of the investigation. Similarly, in a study performed by Varona et al. in Spain, 67% of seropositive HCWs had no previous diagnosis of SARS-CoV-2 infection before serodiagnosis [14]. Our study showed a significant association between the antibody screening and the history of SARS-CoV-2 infection among HCWs, suggesting that HCWs previously infected with SARS-CoV-2 are more likely to be seropositive, which is in accordance with the study performed in New York, where 93% of PCR positive HCWs were also seropositive [11]. Similarly, 40% of seropositive cases in the study performed in Spain did not have a history of SARS-CoV-2 infection, and the same study suggested that those infections might have gone undetected in the diagnostic procedure for SARS-CoV-2 infection [29].

65.9% of seropositive HCWs had flu-like symptoms within the last 6 months. Although the time duration to which the antibodies remain intact following SARS-CoV-2 infection is unclear, the study performed by L'Huillier et al. in Geneva has shown that the antibody titers do not reduce until 6 months after the infection [31]. Studies around the globe, such as a study in Italy [18], a study in Spain [29] and, a study in the UK [19], have shown a significant association between the positive serology and the presence of symptoms. Body ache and headache were the most common symptoms followed by fever and dry cough among the seropositive HCWs in this study. A study performed in a large population of HCWs in the United States has reported the presence of at least one symptom among fever, cough, and dyspnea in a COVID-19 infection [32]. Although body ache, headache, and fever were the most common symptoms among the seropositive, anosmia and ageusia were also reported in 26.8% and 24.4% HCWs, respectively. Lombardi et al. in Italy found that anosmia, ageusia, and fever were the most common symptoms among SARS-CoV-2-infected HCWs [33]. Also, Rudberg et al. in Sweden and Gracia Basteiro et al. in Spain have discussed ageusia and anosmia as the most predictive symptoms of COVID-19 infection in their studies [17, 29]. Our study showed a relatively lower percentage of seropositive HCWs showing anosmia and ageusia, which could be described as variation because of subjects' characteristics and geographical variations [18]. We found that 34.1% of seropositive HCWs were asymptomatic until the time of investigation in the last 6 months. Similarly, Gracia Basteiro et al. reported 23.1% seropositive HCWs to be asymptomatic [29]. Asymptomatic infections among HCWs make it difficult to identify the infected ones that could degrade the strategies to control the infection, and therefore, programs for the regular screening of HCWs for COVID-19 are essential in controlling the spread of infection.

We tested 185 HCWs in our study, among which 41 were seropositive, and all of them had a positive contact history with the COVID-infected patient. The positive contact history was significantly associated with the positive serology, which suggests that the HCWs with positive contact history are more likely to be seropositive. It was in accordance with the findings of Rudberg et al., where the HCWs in contact with COVID-19 patients had higher seroprevalence than the HCWs in contact with non-COVID-19 patients [17]. A study performed by Lombardi et al. in Italy also found that the HCWs with a positive history of being in contact with COVID-19-infected patients are more likely to be seropositive [18]. The significant contribution of this study is that it has demonstrated the infection and seroprevalence of healthcare workers after approximately one year of exposure to the hospital environment by different risk groups of HCWs.

This study has some limitations. This study was a time-framed, single-center study with a relatively small size population. At the time of collection, some of the participants had either been infected recently and had not developed an antibody response or some participants had been infected previously but antibody levels had declined, which could have given an underestimated prevalence rate. Furthermore, it could have been better if we could use ELISA or CLIA for antibody testing.

## 5. Conclusion

The seroprevalence among HCWs was found to be 22.2%. The burden of SARS-CoV-2 infection among HCWs seems to be high, and HCWs are at risk of acquiring infection in the workplace. High prevalence among HCWs was explained by the long exposure of HCWs to the SARS-CoV-2 infected patients. The study revealed that symptomatic infections are more likely to develop strong antibodies.

The relatively higher seroprevalence in our study could be because of the lack of programs for the regular screening of HCWs for SARS-CoV-2 infection, long-term exposure of HCWs with the infected cases (symptomatic or asymptomatic), and the lack of proper PPE. Furthermore, this study recommends that programs for the regular screening of HCWs for SARS-CoV-2 infection regardless of symptoms should be implemented in all the healthcare settings that could help in monitoring the transmission dynamics and the evaluation of infection control policies. Also, this state of the problem could highlight the steps to be taken forward to be prepared for such outbreaks, ensuring that the diagnostic ability and PPE supply is adequate.

## Figures and Tables

**Table 1:** Subjects characteristics and group differences.

Characteristics	Antibody positive *N* (%)	Antibody negative *N* (%)	Total *N* (%)	*p* value
Overall	41 (22.2)	144 (77.8)	185	
Age (years)				
Median (IQR)	27 (22–35.5)	28 (24–36)	27 (24–36)	0.766
Age groups				
20–30	26 (63.4)	89 (61.8)	115 (62.1)	
31–40	11 (26.9)	35 (24.3)	46 (24.9)	
41–50	3 (7.3)	16 (11.1)	19 (10.3)	
Above 50	1 (2.4)	4 (2.8)	5 (2.7)	0.906
Gender				
Male	15 (36.6)	37 (25.7)	52 (28.1)	
Female	26 (63.4)	107 (74.3)	133 (71.9)	0.171
Working department				
Administration	7 (17.0)	19 (13.2)	26 (14.1)	
COVID-19 ward	3 (7.3)	15 (10.4)	18 (9.7)	
Emergency	3 (7.3)	13 (9.0)	16 (8.6)	
General ward	5 (12.2)	19 (13.2)	24 (13.0)	
ICU	5 (12.2)	19 (13.2)	24 (13.0)	
Laboratory	9 (22.0)	25 (17.4)	34 (18.4)	
OPD	9 (22.0)	34 (23.6)	43 (23.2)	0.976
Nature of job				
Doctor	6 (14.6)	20 (13.9)	26 (14.1)	
Nurse	16 (39.0)	71 (49.3)	87 (47.0)	
Laboratory professional	8 (19.5)	20 (13.9)	28 (15.1)	
Administrative staff	7 (17.1)	18 (12.5)	25 (13.5)	
Support staff	4 (9.8)	15 (10.4)	19 (10.3)	0.753
Previous COVID-19 history with positive RT-PCR				
Yes	21 (51.2)	18 (12.5)	39 (21.1)	
No	20 (48.8)	126 (87.5)	146 (78.9)	**<0.001**
Presence of flu-like symptoms within 6 months				
Yes	27 (65.9)	21 (14.6)	48 (25.9)	
No	14 (34.1)	123 (85.4)	137 (74.1)	**<0.001**
Contact history				
Yes	41 (100)	118 (81.9)	159 (85.9)	
No	0 (0)	26 (18.1)	26 (14.1)	**0.002**
Underlying illness				
Yes	3 (7.3)	7 (4.9)	10 (5.4)	
No	38 (92.7)	137 (95.1)	175 (94.6)	0.695

**Table 2:** Risk group based on the degree of exposure to COVID-19.

	Antibody screening		Total (*N*)
	Positive	Negative	
High risk	22	91	113
Moderate risk	12	35	47
Low risk	7	18	25
Total (*N*)	41	144	185

## Data Availability

All data generated during this study are presented in this paper and accessible on reasonable request.

## Abbreviations

SARS-CoV-2: Severe acute respiratory syndrome coronavirus 2
COVID-19: Coronavirus disease 2019
WHO: World Health Organization
HCWs: Healthcare workers
MMTH: Manmohan Memorial Medical College and Teaching Hospital
SPSS: Statistical Package for Social Sciences
IQR: Interquartile range
ELISA: Enzyme-linked immuno-sorbent assay
CLIA: Chemiluminescence immunoassay
PPE: Personal protective equipment.
